# Managing Incidental Genomic Findings in Clinical Trials: Fulfillment of the Principle of Justice

**DOI:** 10.1371/journal.pmed.1001584

**Published:** 2014-01-14

**Authors:** Rafael Dal-Ré, Nicholas Katsanis, Sara Katsanis, Lisa S. Parker, Carmen Ayuso

**Affiliations:** 1 Clinical Research Program, Pasqual Maragall Foundation, Barcelona, Spain; 2 Center for Human Disease Modeling, Department of Cell Biology, Department of Medicine, Duke University, Durham, North Carolina, United States of America; 3 Duke Institute for Genome Sciences and Policy, Duke University, Durham, North Carolina, United States of America; 4 Center for Bioethics and Health Law, University of Pittsburgh, Pittsburgh, Pennsylvania, United States of America; 5 Department of Genetics, Health Research Institute–Jimenez Diaz Foundation University Hospital (IIS-FJD), Madrid, Spain; 6 CIBERER (Centro de Investigación Biomédica en Red de Enfermedades Raras), Instituto de Salud Carlos III, Madrid, Spain

## Abstract

Rafael Dal-Ré and colleagues discuss how incidental findings are likely to be viewed as potential benefits of research participation in genomics trials, and investigators should implement mechanisms to ensure provision of timely and appropriate care. Ensuring provision of such interventions in countries lacking a universal public health care system may prove challenging.

*Please see later in the article for the Editors' Summary*

**Summary Points**

- Genome/exome data are likely to play an increasing role in clinical trials, and incidental findings are likely to be viewed as potential benefits for individuals of research participation.
- Participants in clinical trials across differing trial sites utilizing genome/exome sequencing information should be afforded the same standard of care, including return of incidental genomic findings.
- Participants may opt in to receiving incidental genomic findings, and clinical trial investigators should implement mechanisms to ensure provision of timely and appropriate care to prevent or ameliorate conditions associated with incidental findings.
- Ensuring the provision of such interventions in countries lacking a universal public health-care system may prove more challenging than in countries with public health-care support.

Three basic principles are key in assessing the ethics of any research conducted on humans: respect for persons (or autonomy), beneficence, and justice [1]. Respect for persons entails individuals being afforded the right to decide what should happen to them; this principle is fulfilled through a valid informed consent process. Research participants should expect investigators to make all efforts to secure their well-being, i.e., far beyond the “do not harm” Hippocratic maxim: beneficence refers to ensuring a favorable benefit/risk assessment of the proposed research. The principle of justice requires that research's benefits and burdens be distributed fairly, and that research avoids the injustice resulting “when some benefit to which a person is entitled is denied without good reason” [1]. Initially understood as a principle to protect vulnerable people from the risks of clinical research, since the 1980s, when HIV/AIDS patients drew attention to the potential medical benefit of enrolling in clinical trials, this principle is understood also to encompass fair access to the potential benefits of research participation [2]. Thus, in addition to altruistic reasons, some participants enroll in clinical trials with the hope, or even expectation, that participation offers an opportunity to benefit through treatment, medical care, and disease monitoring to which, in some circumstances, the participants may otherwise lack access.

Increasingly, clinical trials to develop new drugs and biologics involve whole genome or exome sequencing (WGS/WES), including for biomarker characterization, for identification of genomic risk factors, and for population-based research [3]. WGS/WES by nature produces incidental genomic findings, i.e., findings that have potential health or reproductive importance discovered in the course of conducting research but beyond the aims of the study [4]. In determining how to manage incidental genomic findings in clinical trials, we suggest two themes for consideration: (1) the maintenance of clinical standards of care for WGS/WES and (2) the obligation of investigators to manage trial participants fairly.

In response to growing recognition that actionable incidental genomic findings could be of value for patient care, the American College of Medical Genetics and Genomics (ACMG) published recommendations for management of incidental genomic findings obtained in clinical practice [5]. The ACMG recommends that clinical genome sequencing laboratories actively seek and report pathogenic variants identified in 56 genes associated with 24 conditions, all with evidence that early intervention can prevent or ameliorate severe adverse medical outcomes [5],[6] (Box 1). The appropriate approach to handling incidental genomic findings in the clinical context is under substantial debate [7]–[12]. Relevant sets of recommendations are to be issued in due course [13],[14]. Nevertheless, it is reasonable to assume that a standard of care will emerge for returning incidental genomic findings to patients receiving WGS/WES in clinical contexts. In anticipation of this eventuality, it will be important for stakeholders to consider the relevance of such a standard of care for ethical clinical trial design and conduct.

**Box 1. Examples of Genes and Associated Diseases/Conditions Recommended by the American College of Medical Genetics and Genomics for Reporting of Incidental Findings in Clinical Exome and Genome Sequencing [5]: Age of Onset and Prevention Strategies**

**Example 1** [**29**]

- **Genes/diseases:** *BRCA1*, *BRCA2*/breast and ovarian cancers
- **Age of onset:** Breast cancer, ≥25 years old; ovarian cancer, ≥40 years old
- **Prevention interventions for women:**
  - Breast cancer: <25 years old: annual clinical breast exam; >25 years old: surveillance (annual mammography and magnetic resonance imaging); clinical breast exam (every six months) or prophylactic bilateral mastectomy; chemoprevention: not proven
  - Ovarian cancer: 30–35 years old: periodic screening (blood test for CA-125 and transvaginal ultrasonography); >35 years old: prophylactic bilateral salpingo-oophorectomy

**Example 2** [**30**]

- **Genes/diseases:** *PKP2*, *DSP*, *DSC2*, *TMEM43*, *DSG2*/arrhythmogenic right ventricular cardiomyopathy/dysplasia
- **Age of onset:** Usually from adolescence onwards (4–64 years old)
- **Prevention interventions:**
  - Prevention of sudden cardiac death by dramatically reducing exercise and discontinuing competitive athletics
  - Prevention or delay of disease progression: beta-blockers and angiotensin-converting enzyme inhibitors
  - Electrocardiogram monitoring (to detect sustained ventricular tachycardia, arrythmogenic syncope, or frequent ventricular ectopy and/or non-sustained ventricular tachycardia)

**Example 3** [**31**]

- **Genes/diseases:** *LDLR*, *APOB*, *PCSK9*/familial hypercholesterolemia
- **Age of onset:** homozygous: adolescence; heterozygous: men—heart attacks in ≥40 s (85% had one by age 60); women—heart attacks in ≥50 s
- **Prevention interventions:**
  - Children and adolescents: diet
  - Children and adults: lifestyle interventions (diet, physical activity, no smoking)
  - Drugs to be considered (e.g., for elevated low-density lipoprotein or other risk factors): statins, bile and sequestrant resins, niacin, ezetemibe, gemfibrozil, fenofibrate, and others

## Returning Incidental Genomic Findings in Clinical Trials

Employing WGS/WES in clinical trials could advance the development of new treatments and diagnostic tools for many disorders and conditions, for instance, in oncology [15]. However, ethical and regulatory issues regarding the management of incidental genomic findings should be addressed before WGS/WES is performed routinely in therapeutic trials. If we assume that the deliberate search for genetic variants will become the standard of care in the clinical setting, it is reasonable to expect the same standards to be applied to participants in clinical trials.

In recent years, the idea that investigators have “ancillary care” obligations to their study participants has emerged [16]. Ancillary care exceeds the demands of the particular study interventions, sound scientific practice, participant safety, and response to adverse events. Although ancillary care is sometimes considered to be owed as a matter of justice or beneficence, the obligation to provide such care is grounded ethically in the investigator–participant relationship and the special permissions granted to investigators by participants during informed consent [17]. Following the ancillary care framework [16],[18], it seems appropriate to apply a standard-of-care list of genes for which to actively seek pathogenic variants in clinical research. If, as standard of care, a patient could be informed of particular variants reported as actionable incidental genomic findings when visiting a physician, it is reasonable to argue that the same patient, as a participant in a trial involving WGS/WES, may have the right to be informed about those same incidental genomic findings.

Although the potential benefit associated with identification of an incidental genomic finding resides largely in its informational value, for individuals to receive the full benefit of learning of an incidental genomic finding, subsequent preventive interventions would need to be undertaken to prevent or ameliorate the manifestation of the associated condition. Ensuring that this benefit is distributed fairly to all participants in a clinical trial involving WGS/WES presents particular challenges.

## Management of Trial Participants' Care following Disclosure of Incidental Genomic Findings

Because justice demands that similar people be treated similarly and that irrelevant differences between them should not result in differential benefit [1], justice in clinical trials requires that participants have equitable access to potential benefits of research, including the benefits that may result from learning of an incidental genomic finding. Since learning of incidental genomic findings identified by WGS/WES is a potential benefit of study participation—as indeed it is, if access to such information is considered standard of care in the clinical care context—then this opportunity should be afforded to all study participants, regardless of differences, such as their personal ability to afford medical care for the condition associated with the incidental genomic finding or their access to public universal health care.

If the standard of care for clinical WGS/WES evolves to include actively seeking a list of genetic variants associated with actionable medical conditions, then participants in clinical trials involving WGS/WES may justifiably expect to receive sequencing information to promote their health [19]. Given that research protocols require the ethically appropriate conduct of clinical trials across all sites and countries, all participants in a given trial, irrespective of the different health-care systems of the localities involved, should have access to the same (or similar) patient care procedures and facilities, including appropriate incidental genomic finding management.

Varying types of health-care systems at trial sites could present a significant challenge with regards to the health-care delivery response to incidental genomic findings. If an incidental genomic finding is to be reported to a trial participant, the investigator would be responsible for provision of comprehensive counseling including discussion of available intervention choices. While, ideally, all treatment and care choices resulting from participation would be provided free of charge to a trial participant, in reality, access to interventions will vary based on the clinical trial site. If the country where the trial is conducted has a universal public health-care system, it would seem reasonable that the public system would provide treatment in advance of disease manifestation. This is reasonable since if the trial participant had not undergone the WGS/WES, and hence had not received any intervention to prevent the development of the disease, the public health care system eventually would have had to address treatment of the disease when it manifested.

However, in regions lacking a universal public health-care system, a clinical trial sponsor could justifiably be held responsible for implementing mechanisms to ensure the provision of the interventions needed by all participants facing medically actionable incidental genomic findings. Institutional Review Boards/Research Ethics Committees (IRB/RECs) would be expected to approve trial protocols with WGS/WES only if all potential participants are assured in the informed consent process that any incidental genomic findings will be properly managed, as per the standard of care. Although important in any trial, this is especially relevant in international clinical trials, where the sponsor has an obligation to fulfill the requirements of the principle of justice across all countries and settings. Inadequate intervention in response to incidental genomic findings resulting from WGS/WES should not be a means of discrimination among trial participants within the same or across different countries. Relevant IRB/RECs should review the mechanisms of providing the appropriate standard of care and the nature, duration, and limits of interventions to be provided.

## Maintenance of Justice in Multicenter Clinical Trials

Because most conditions associated with actionable genetic variants (such as those listed by the ACMG) will require long-term management, sponsors could be tempted to conduct trials involving constitutional WGS/WES only in countries with universal public health-care systems. This would have an unethical discriminatory effect, both depriving members of some countries or communities of the potential benefits of clinical trial participation and failing to demonstrate the relevance of trial outcomes for members of those populations. To avoid this potential injustice, sponsors should consider securing a health-care insurance agreement for trial participants to assure access to the interventions needed to manage any reported incidental genomic findings.

All participants in clinical trials, including international trials, have the right to be treated with the standard of care. In countries—including low- or middle-income countries—where the standard of care may be very different from that of a wealthy country (or may not even exist), it has been suggested in other circumstances [20] that the external sponsor should reach an agreement with the IRB/REC of its own country and with the trial investigators, the health authorities, and the IRB/REC of the site (host) country regarding mechanisms to coordinate appropriate care in order to maintain justice.

Finally, some commentators [21] have suggested that concern for facing liability for nondisclosure of actionable incidental genomic findings may prompt some United States–based clinical investigators to actively search for ACMG-recommended incidental genomic findings to avoid negligence-based malpractice [22]. In other countries, however, investigators may not feel legally motivated to search for incidental genomic findings in their trial participants. The different legal climates of trial sites could be another source of discrimination among participants in the same international trial that could be prevented by asking all site investigators to adhere to the same standard for incidental genomic finding searches.

## Further Reflections on Returning Incidental Genomic Findings

The number of trial participants likely to be affected is relevant for estimating the likely cost of managing incidental genomic findings as an obligation of ancillary care. One (or more) mutations—if the ACMG list would be applied—is presumed to be present in 1% of all trial participants [5]. This would mean that, for instance, since 75% of all oncology trials recruit no more than 100 patients [23], the expected (mean) number of patients with incidental genomic findings would be one per trial.

The ACMG recommendation that incidental genomic findings be reported regardless of a patient's preference regarding whether to receive this information [5] has been rejected on the grounds that patients have a right “not to know” genomic information and that imposing such findings would fail to respect patient autonomy [7],[24]. The ACMG recommendations also have been challenged as scientifically premature [25] and are controversial with regard to incidental genomic findings discovered in children [25],[26].

Emerging data show that some individuals want to receive all types of WGS/WES information—including those genomic results that cannot be interpreted [19]. We believe that as a general rule it is required ethically only to inform research participants who have opted to receive information of any actionable incidental genomic finding that can be medically managed [4]. Whether the international community endorses the ACMG list or a similar one for routine clinical practice, in effect establishing a standard of care, we propose that therapeutic trials should incorporate a similar standardized approach to reporting incidental genomic findings found in WGS/WES as part of a trial.

We feel that in a research context, participants' autonomous decision not to know genomic information should be respected. To respect the autonomy of clinical trial participants, explanation of the possibility that WGS/WES will result in incidental genomic findings should be disclosed as part of the consent process. Participants should receive comprehensive pre-sequencing counseling and be informed of the options to learn or not learn of any resulting incidental genomic finding. They should also have the option of changing their minds with regard to learning of incidental genomic findings at any point during the trial [7],[27], prior to actual disclosure of an identified incidental genomic finding. Furthermore, to ensure requisite validity of the finding, upon identification of an incidental genomic finding that is a candidate for disclosure (i.e., one that is to be offered according to the prevailing clinical standard of care), the incidental genomic finding should be confirmed in accordance with clinical laboratory standards and regulations [28].

## Conclusion

In anticipation of genome sequencing becoming commonplace in clinical trials, from the beginning, the scientific community should be respectful of the rights of all participants, regardless of where they are recruited. Among these rights is the right to receive the standard of care regarding reporting of incidental genomic findings and provision of interventions necessary to prevent or ameliorate medical conditions associated with those findings. The challenges of developing a common approach for clinical trials involving WGS/WES warrant an open debate among stakeholders (funding organizations, investigators, regulators, IRB/REC members, bioethicists, and patient advocates) in the clinical research enterprise.

## Supporting Information

Alternative Language Summary Points S1 **Spanish translation of the Summary Points by R D-R.**
(DOCX)

## Abbreviations

ACMG: American College of Medical Genetics and Genomics
IRB/REC: Institutional Review Board/Research Ethics Committee
WGS/WES: whole genome or exome sequencing
