# Supplementary material for: Managing Incidental Genomic Findings in Clinical Trials: Fulfillment of the Principle of Justice
Source: PLoS Med. 2014 Jan 14;11(1):e1001584. doi: 10.1371/journal.pmed.1001584 (PMC3891615; doi:10.1371/journal.pmed.1001584)
Supplement: Alternative Language Summary Points S1 — Spanish translation of the Summary Points by R D-R. (DOCX) [file pmed.1001584.s001.docx]

*Summary points: Spanish version*

-Probablemente, los datos obtenidos por secuenciación del genoma/exoma representarán un papel de importancia creciente en los ensayos clínicos; es probable que los hallazgos inesperados sean contemplados como un beneficio potencial para los participantes

-Los diferentes centros participantes en ensayos clínicos que utilicen información de secuenciación del genoma/exoma deben ofrecer la misma asistencia médica estándar a todos los participantes, incluyendo la relativa a los hallazgos genómicos inesperados

-Los participantes pueden aceptar ser informados de los hallazgos genómicos inesperados, por lo que los investigadores deben implementar mecanismos que aseguren la provisión puntual y adecuada de la asistencia que prevenga o aminore el trastorno asociado al hallazgo inesperado

- Asegurar la provisión de tales intervenciones en países que carecen de un sistema público de salud universal, puede ser más difícil que en los países que cuentan con él.
